# Non-invasive computed tomography coronary angiography as a gatekeeper for invasive coronary angiography

**DOI:** 10.1007/s10554-012-0059-8

**Published:** 2012-05-11

**Authors:** Fleur R. de Graaf, Joëlla E. van Velzen, Stephanie M. de Boer, Jacob M. van Werkhoven, Lucia J. Kroft, Albert de Roos, Allard Sieders, Greetje J. de Grooth, J. Wouter Jukema, Joanne D. Schuijf, Jeroen J. Bax, Martin J. Schalij, Ernst E. van der Wall

**Affiliations:** 1 Department of Cardiology, Leiden University Medical Center, Albinusdreef 2, 2333 ZA Leiden, The Netherlands; 2 The Interuniversity Cardiology Institute of the Netherlands, Utrecht, The Netherlands; 3 Department of Radiology, Leiden University Medical Center, Leiden, The Netherlands; 4 Department of Cardiology, Rijnland Hospital, Leiderdorp, The Netherlands

**Keywords:** Cardiac imaging, Coronary artery disease, Multidetector computed tomography, Invasive coronary angiography

## Abstract

To determine the rate of subsequent invasive coronary angiography (ICA) and revascularization in relation to computed tomography coronary angiography (CTA) results. In addition, independent determinants of subsequent ICA and revascularization were evaluated. CTA studies were performed using a 64-row (n = 413) or 320-row (n = 224) multidetector scanner. The presence and severity of CAD were determined on CTA. Following CTA, patients were followed up for 1 year for the occurrence of ICA and revascularization. A total of 637 patients (296 male, 56 ± 12 years) were enrolled and 578 CTA investigations were available for analysis. In patients with significant CAD on CTA, subsequent ICA rate was 76  %. Among patients with non-significant CAD on CTA, subsequent ICA rate was 20 % and among patients with normal CTA results, subsequent ICA rate was 5.7 % (*p* < 0.001). Of patients with significant CAD on CTA, revascularization rate was 47 %, as compared to a revascularization rate of 0.6 % in patients with non-significant CAD on CTA and no revascularizations in patients with a normal CTA results (*p* < 0.001). Significant CAD on CTA and significant three-vessel or left main disease on CTA were identified as the strongest independent predictors of ICA and revascularization. CTA results are strong and independent determinants of subsequent ICA and revascularization. Consequently, CTA has the potential to serve as a gatekeeper for ICA to identify patients who are most likely to benefit from revascularization and exclude patients who can safely avoid ICA.

## Introduction

Invasive coronary angiography (ICA) is routinely used for the identification of patients with suspected coronary artery disease (CAD). Advantages of ICA are high resolution imaging and the possibility of revascularization by percutaneous coronary intervention (PCI). Due to its invasive nature, ICA is associated with a small risk of complications, radiation exposure and relatively high cost of hospital stay. Additionally, the rate of normal ICA examinations is still quite high and health-care costs associated with the increase in ICA and revascularization rates are substantial. Moreover, a recent multicenter study showed that PCI has no superiority over pharmacological therapy in patients with stable CAD [1]. Accordingly a non-invasive test to select the most suitable patients for ICA and revascularization would be preferable. Most traditional non-invasive cardiac imaging techniques rely on the detection of stress-inducible ischemia [2]. However, with the introduction of computed tomography coronary angiography (CTA), the non-invasive anatomic assessment of CAD with high diagnostic accuracy has become possible. Prior studies have shown that CTA allows reliable patient risk stratification, and normal CTA examinations indicate good prognosis [3, 4]. Although CTA cannot replace ICA, this technique could serve as a gatekeeper for ICA in selected patients, and thus avoid unnecessary additional examinations. At the same time concerns have been raised that CTA may trigger unnecessary referral for ICA. Rates of ICA and interventional therapy following CTA have been largely unreported. The purpose of the present study therefore was to determine the rate of subsequent ICA and revascularization in relation to CTA results. Furthermore, independent determinants of subsequent ICA and revascularization were investigated.

## Methods

### Patient population

The study group consisted of patients who were referred for CTA as part of a large ongoing registry exploring the prognostic value of CTA [5]. Reasons for referral were typical chest pain, atypical chest pain and non-anginal chest pain, according to the appropriate use criteria for cardiac computed tomography [6]. Exclusion criteria for CTA investigation were: renal insufficiency (glomerular filtration rate <30 ml/min), (supra)ventricular arrhythmias, known allergy to iodine contrast material, severe claustrophobia, pregnancy and high heart rate in the presence of contraindications to β-blocker medication [7]. Patients were entered prospectively into the departmental patient information system (EPD-Vision^®^, Leiden University Medical Center) and retrospectively analysed. Patients with known CAD or congenital cardiac abnormalities were excluded from the study.

### CTA data acquisition

CTA studies were performed using a 64-row (n = 413) or 320-row (n = 224) multidetector scanner (Aquilion 64, and Aquilion ONE, Toshiba Medical Systems, Otawara, Japan) with 64 and 320 simultaneous detector rows, respectively (each 0.5 mm wide), as previously described [8, 9]. One hour before the investigation, oral β-blocker medication (metoprolol 50 or 100 mg) was administered to patients with a heart rate ≥65 beats/min, unless contra-indicated. The total amount of non-ionic contrast media (Iomeron 400; Bracco, Milan, Italy) injected into the antecubital vein was 60–100 ml (depending on scanner type and body weight) at a flow rate of 5.0–6.0 ml/s. In order to synchronize the arrival of the contrast media, bolus arrival was detected using a real-time bolus tracking technique. All images were acquired during a single inspiratory breath-hold of maximally 12 s for 64 row-CTA and 5 s for 320-row CTA. For 64-row CTA, a helical-scanning technique was used as previously described [10]. In brief, during the examination the ECG was registered simultaneously for retrospective gating of the data. A collimation of 64 × 0.5 mm was used. During 320-row CTA, the ECG was registered simultaneously for prospective triggering of the data. A collimation of 320 × 0.5 mm was used and the entire heart was imaged in a single heart beat, as previously reported [11].

The estimated mean radiation dose for 64-row CTA was 18.1 ± 5.9 mSv in patients scanned using retrospective ECG gating. The estimated mean radiation dose for 320-row CTA was 3.2 ± 1.1 mSv if scanned ful-dose at 75 % of the cardiac cycle. In patients who were scanned full-dose at 65–85 % of the R–R interval, estimated mean radiation dose was 7.1 ± 1.7 mSv.

### CTA data analysis

Data were transferred to a remote workstation with dedicated analysis software (for 64-row CTA reconstructions: Vitrea 2; for 320-row CTA reconstructions: Vitrea FX 2.0, Vital Images, Minnetonka, MN, USA). First, calcium score was assessed and an overall Agatston score was registered for each patient. Next, coronary arteries were evaluated as previously described [8]. Presence of CAD was assessed as recommended by the SCCT guidelines for the interpretation and reporting of CTA [12]. Each scan classified as having (1) normal, (2) non-significant CAD (luminal narrowing <50 % in diameter), (3) obstructive CAD (≥50 % luminal narrowing), as described [13]. In addition, the presence of significant left main disease and significant three-vessel disease was noted. After data evaluation, CTA results were entered in into the departmental Cardiology Information System (EPD-Vision^®^) without recommendations for further clinical management. Further clinical management was determined at the discretion of the referring cardiologist.

### ICA and revascularization

ICA was performed according to standard techniques. Following CTA, patients were followed up for 1 year for the occurrence of ICA and revascularization. Patient follow-up information was obtained by one observer, blinded to the baseline CTA results, using data from clinical visits and/or standardized telephone interviews.

### Statistical analysis

Statistical analysis was performed using SPSS software (version 16.0, Inc., Chicago, Illinois). Quantitative data were expressed as mean ± standard deviation (SD). Categorical variables were described as numbers and percentages and comparison was performed by Chi-square test. Univariate analysis of clinical baseline variables and significant CAD on CTA was performed. For each variable, odds ratio (OR) and 95 %-confidence interval (CI) were calculated. Subsequently, multivariate logistic regression analysis for ICA and revascularization were performed (using backward elimination method with *p*-value >0.2 as the criterion for elimination) to determine the independent association with significant CAD on CTA and significant three-vessel or left main disease on CTA, each corrected for clinical baseline variables (age, gender, diabetes, hypercholesterolemia, hypertension, family, smoking and obesity) in a separate model. A *p* value < 0.05 was considered statistically significant.

## Results

### Study population

A total of 637 patients were enrolled in the study population. An overview of the patient characteristics is shown in Table 1. In brief, 47 % of patients were male with a mean age of 56 ± 12 years. Reasons for referral were typical chest pain in 21 %, atypical chest pain in 46 % and non-anginal chest pain in 33 %.

**Table 1:** Clinical characteristics (n = 637)

Age (years)	56 ± 12
Men/women	296/341
Diabetes	19 %
Hypercholesterolemia^a^	34 %
Hypertension^b^	43 %
Family history of CAD^c^	46 %
Smoking	20 %
Obesity^d^	21 %
Reason of referral for CTA	
Typical chest pain	21 %
Atypical chest pain	46 %
Non-anginal chest pain	33 %

Data are absolute values, percentages or means ± standard deviation

*BMI* body mass index, *CAD* coronary artery disease, *CTA* computed tomography coronary angiography

^a^Serum total cholesterol ≥230 mg/dl and/or serum triglycerides ≥200 mg/dl or treatment with lipid lowering drugs, ^b^ Defined as systolic blood pressure ≥140 mm Hg and/or diastolic blood pressure ≥90 mm Hg and/or the use of antihypertensive medication, ^c^ Defined as presence of coronary artery disease in first degree family members at <55 years in men and <65 years in women, ^d^ Defined as a BMI ≥30 kg/m^2^

A total of 27 scans (4.2 %) were of non-diagnostic image quality, and excluded from the analysis. The presence of blooming artifacts in patients with a high calcium score ≥400 accounted for 7 uninterpretable scans. Furthermore, 30 patients (3.8 %) were lost to follow-up and 2 patients died before follow up was completed. As a result, a total of 578 patients were included in the analysis.

### CTA results

In a total of 578 patients, CTA results were normal in 212 patients (37 %), non-significant CAD was observed in 177 patients (30 %) and significant (≥50 %) CAD was identified in 189 patients (33 %). Additionally, significant three-vessel or left main disease on CTA was observed in 34 patients (5.9 %), while the presence of significant three-vessel or left main disease could not be determined in two patients due to insufficient image quality.

### ICA

**Fig. 1:**
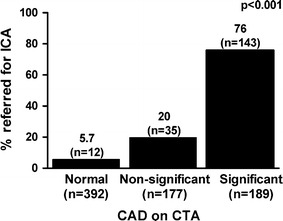
*Bar graph* illustrating the relationship between degree of CAD on CTA and subsequent referral for ICA. *CAD* coronary artery disease, *CTA* computed tomography coronary angiography, *ICA* invasive coronary angiography

Subsequent to CTA, ICA was performed in 190 patients (33 %). The mean duration between CTA and ICA was 2.6 ± 2.7 months. Of the 189 CTA investigations with significant CAD, subsequent ICA rate was 76 % (n = 143). Among 177 patients with non-significant CAD on CTA, subsequent ICA rate was 20 % (n = 35) and among 212 patients with normal CTA results, subsequent ICA rate was 5.7 % (n = 12; *p* < 0.001). Figure 1 illustrates the relationship between CTA results and subsequent ICA. Moreover, of the 34 patients with significant three-vessel or left main disease on CTA, subsequent ICA rate was 88 % (n = 30), while ICA rate in 542 patients without significant three-vessel or left main disease on CTA was 29 % (n = 158, *p* < 0.001).

Univariate regression analysis was performed to identify determinants of subsequent ICA. Table 2 shows that significant CAD on CTA (OR 22.62) as well as significant three-vessel or left main disease on CTA (OR 18.23) were identified as a significant univariate determinant of subsequent ICA. Furthermore, the clinical baseline variables age, gender, hypercholesterolemia, hypertension and smoking were significant univariate determinants of ICA.

**Table 2:** Independent determinants of subsequent ICA and revascularization

Variable	Univariate		Multivariate	
	OR (95 % CI)	*p*-value	OR (95 % CI)	*p*-value
ICA				
Age	1.05 (1.03–1.06)	<0.001	1.02 (0.99–1.04)	0.112
Gender	1.92 (1.35–2.73)	<0.001	1.81 (1.13–2.91)	0.014
Diabetes	1.35 (0.87–2.08)	0.182	–	–
Hypercholesterolemia	2.19 (1.53–3.14)	<0.001	1.42 (0.87–2.30)	0.162
Hypertension	2.09 (1.47–2.98)	<0.001	1.51 (0.93–2.46)	0.098
Family history of CAD	0.83 (0.58–1.17)	0.282	–	–
Smoking	2.70 (1.78–4.09)	<0.001	2.35 (1.33–4.14)	0.003
Obesity	1.08 (0.69–1.67)	0.749	–	–
Significant CAD on CTA^a^	22.62 (14.41–35.51)	<0.001	18.60 (11.46–30.19)	<0.001
Significant three-vessel or left main disease on CTA^a^	18.23 (6.32–52.59)	<0.001	15.67 (4.59–53.43)	<0.001
Revascularization				
Age	1.05 (1.03–1.07)	<0.001	1.02 (0.99–1.06)	0.134
Gender	2.80 (1.73–4.53)	<0.001	2.90 (1.54–5.46)	0.001
Diabetes	2.08 (1.24–3.49)	0.005	2.10 (1.00–4.43)	0.050
Hypercholesterolemia	2.31 (1.46–3.66)	<0.001	1.45 (0.78–2.69)	0.243
Hypertension	1.92 (1.22–3.04)	0.005	–	–
Family history of CAD	0.67 (0.42–1.07)	0.095	–	–
Smoking	3.43 (2.11–5.58)	<0.001	3.24 (1.60–6.57)	0.001
Obesity	1.09 (0.62–1.92)	0.773	–	–
Significant CAD on CTA^a^	338.06 (46.53–2,456.30)	<0.001	282.61 (38.21–2,090.31)	<0.001
Significant three-vessel or left main disease on CTA^a^	15.62 (7.27–33.54)	<0.001	12.31 (5.52–28.91)	<0.001

*CAD* coronary artery disease, *CTA* computed tomography coronary angiography, *ICA* invasive coronary angiography

^a^Each variable was included in a separate model corrected for clinical baseline variables (age, gender, diabetes, hypercholesterolemia, hypertension, family, smoking and obesity). Results from multivariate analysis for clinical baseline variables shown in the table were derived from the model including significant CAD on CTA

Subsequently, multivariate logistic regression analysis for ICA was performed to determine the independent association with significant CAD on CTA and significant three-vessel or left main disease on CTA, each corrected for clinical baseline variables in a separate model. Significant CAD on CTA (OR 18.60) and significant three-vessel or left main disease on CTA (OR 15.67) were identified as the strongest independent predictors of ICA. Other determinants of ICA of lesser statistical significance were gender and smoking. Table 2 shows the results of uni- and multivariate regression analysis to identify determinants of subsequent ICA.

### Revascularization

**Fig. 2:**
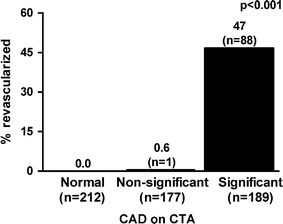
*Bar graph* illustrating the relationship between degree of CAD on CTA and revascularization. *CAD* coronary artery disease, *CTA* computed tomography coronary angiography

A total of 89 patients (15 %) underwent revascularization, of whom 74 patients underwent PCI and 15 patients coronary artery bypass grafting (CABG). Of the 189 patients with significant CAD on CTA, revascularization rate was 47 % (n = 88), as compared to a revascularization rate of 0.6 % (n = 1) in 348 patients with non-significant CAD on CTA. Of note, this patient had a significant lesion in the distal RCA, which was underestimated on CTA. No revascularizations were performed in patients with a normal CTA examination (*p* < 0.001). The frequency of revascularization in relation to CAD on CTA is illustrated in Fig. 2.

In 34 patients with significant three-vessel or left main disease on CTA, revascularization rate was 68 % (n = 23), as compared to 12 % (n = 64) in 542 patients without significant three-vessel or left main disease on CTA (*p* < 0.001). Table 2 shows that significant CAD on CTA (OR 338.06) as well as significant three-vessel or left main disease on CTA (OR 15.62) were identified as significant determinants of revascularization in univariate analysis. Furthermore, the clinical baseline variables age, gender, hypercholesterolemia, hypertension and smoking were significant univariate determinants of revascularization.

Next, multivariate logistic regression analysis for revascularization was performed to determine the independent association of significant CAD on CTA and significant three-vessel or left main disease on CTA, each corrected for clinical baseline variables in a separate model. Multivariate regression analysis identified significant CAD on CTA (OR 282.61) and significant three-vessel or left main disease on CTA (OR 12.31) as the strongest predictors of revascularization. Additional significant determinants were gender and smoking. In Table 2, the results of uni- and multivariate regression analysis to identify determinants of revascularization are shown.

## Discussion

The present clinical investigation evaluated the association between CTA results and subsequent rates of ICA and revascularization. The majority of patients with significant CAD on CTA were referred for subsequent ICA (76 %), while in patients with normal CTA results a very low rate of referral was demonstrated (5.7 %). Additionally, no patients with normal CTA results underwent revascularization. Moreover, significant CAD and significant three-vessel or left main disease on CTA were identified as the strongest independent determinants of subsequent ICA and revascularization.

### Previous literature

The use of CTA to reliably exclude significant CAD is supported by extensive literature validating this technique against ICA [14]. Nevertheless, limited information is available regarding the influence of CTA results on clinical decision making and referral for downstream testing such as ICA. Henneman and colleagues previously showed that a substantial proportion of patients with suspected CAD have normal coronaries on CTA examination [15]. As a result, in a substantial percentage of patients with suspected CAD, significant stenosis may be excluded using CTA. Furthermore, Chow et al. [16] recently studied the clinical impact of CTA on the rate of normal ICA. In a large cohort of 7,017 consecutive patients who were referred for ICA before and after implementation of a dedicated CTA program, the implementation of CTA had a positive effect on ICA referral by reducing the frequency of normal ICA from 32 to 27 %. The present results expand on these findings, in identifying a strong association between CTA results and referral for ICA. Moreover, the current findings showed a high percentage of normal and non-significant CT results. Considering that normal CTA examinations are associated with a good prognosis [17], these data imply that, using CTA, a large proportion of patients with chest pain or a high risk profile may be safely excluded from ICA.

Even though significant CAD on CTA was the strongest predictor for revascularization, still a considerable proportion of patients (24 %) with significant CTA results were not referred for ICA. Similarly, a small percentage of patients with non-significant and normal CTA results (20 and 5.7 %, respectively) were referred for ICA. These findings could be explained by the fact that other clinical information and test results, such as exercise ECG or myocardial perfusion imaging (MPI), may have also influenced referral for ICA. Indeed, clinical presentation and functional information also influence subsequent referral to ICA and revascularization. While no previous studies have investigated ICA rates in relation to CTA results, a prior investigation by Bateman and colleagues showed comparable ICA referral rates in patients who were referred for MPI using single photon emission computed tomography (SPECT) [18]. In a group of 4,162 patients with a mean follow up of 8.9 months, 60 % of patients with high-risk ischemia were referred for ICA, as compared with 9 % with mild ischemia and 3.5 % of patients without ischemia on SPECT. In this population, 40 % of high-risk patients were not referred for invasive imaging, most likely due to the fact that other clinical information and previous study results also influenced patient management. A more recent study by Shaw et al. [19] showed comparable results. In analyzing post-SPECT referral rates, 52 % of patients with 3 ischemic perfusion areas underwent ICA. Unfortunately, studies directly comparing CTA and MPI are not available, and future investigations are warranted.

### Anatomical and functional imaging prior to ICA

Most traditional non-invasive cardiac imaging techniques rely on the detection of stress-inducible ischemia [18, 20, 21]. In this setting, perfusion abnormalities or systolic dysfunction serve as surrogate markers for flow-limiting CAD [22]. Although CTA and MPI (the most frequently applied functional imaging technique) provide complementary information [22], concerns about radiation exposure preclude the use of both CTA and MPI in all patients. With the introduction of CTA, the use of MPI as a gatekeeper for ICA has been challenged [23]. First, CTA has a negative predictive value approaching 100 %, making it an excellent modality for the exclusion of CAD in patients with a low-to-intermediate pre-test likelihood. Conversely, MPI enables the identification of perfusion abnormalities, due to which this modality is particularly suitable for ruling in CAD, especially in higher risk patients or patients with unknown CAD [24]. Thus, individual patient characteristics are important in the choice of non-invasive imaging modality to further guide patient management. Second, while both MPI and CTA are associated with radiation exposure, radiation exposure of CTA has been substantially reduced using novel low-dose algorithms. In daily clinical practice, however, the choice of non-invasive imaging modality prior to ICA may also depend on availability [20] and local expertise. Finally, with the large increase in health-care costs focus is increasingly shifting to cost-effective use of resources. Preliminary results suggest that costs of CTA as a gatekeeper for ICA may be significantly lower than MPI [25] and therefore more cost-effective. Nevertheless, precise cost-benefit analyses are currently not available, and further studies evaluating the relationship between CTA and MPI in selecting patients for ICA are warranted.

### Clinical implications

The use of CTA to exclude significant CAD may allow cardiologists to restrict referral for ICA to patients in whom the need for interventional therapy is highly likely [26]. In patients with a normal CTA examination CAD can be safely ruled out and the patient may be reassured. Conversely, patients with significant stenosis on CTA should be referred for further evaluation. Furthermore, patients with recurrent or worsening symptoms as well as patients with left main or three-vessel disease on CTA could be directly referred for ICA. In patients with non-significant stenosis on CTA, however, medical therapy and lifestyle interventions may be appropriate and these patients may be excluded from ICA. Nevertheless, in patients with uncertain results, functional analysis could be performed to further guide referral for ICA. Notably, while CTA may aid risk stratification for the presence of CAD in patients with a low-to-intermediate risk profile, CTA may be less useful in patients with known CAD, in whom the need for ICA and interventional therapy is likely [6, 27, 28].

### Limitations

Several limitations of the present study merit further consideration. Firstly, CTA is inherently associated with ionizing radiation [29]. Secondly, CTA and ICA do not provide information regarding the functional significance of a lesion. Combined anatomic and perfusion imaging using either a hybrid imaging approach or volumetric CTA in a single examination would be advantageous and research is ongoing [30]. Third, the effect of other clinical information, such as perfusion imaging, may have also influenced referral for ICA. However, studying the effects other tests as well as cost-benefit analysis were beyond the scope of this study. Last, the present investigation did not evaluate clinical outcome. Future studies are needed to evaluate the effect of CTA on clinical outcome and health-care costs.

## Conclusion

The present investigation showed that the results of CTA are strong and independent determinants of subsequent ICA as well as revascularization. Consequently, CTA has the potential to serve as a gatekeeper for ICA to identify patients who are most likely to benefit from revascularization and exclude patients who can safely avoid ICA.

## References

[1] Boden WE, O’Rourke RA, Teo KK (2007). Optimal medical therapy with or without PCI for stable coronary disease. N Engl J Med.

[2] Hachamovitch R, Hayes SW, Friedman JD (2004). Stress myocardial perfusion single-photon emission computed tomography is clinically effective and cost effective in risk stratification of patients with a high likelihood of coronary artery disease (CAD) but no known CAD. J Am Coll Cardiol.

[3] Min JK, Shaw LJ, Devereux RB (2007). Prognostic value of multidetector coronary computed tomographic angiography for prediction of all-cause mortality. J Am Coll Cardiol.

[4] van Velzen JE, de Graaf FR, Kroft LJ et al (2011) Performance and efficacy of 320-row computed tomography coronary angiography in patients presenting with acute chest pain: results from a clinical registry. Int J Cardiovasc Imaging 21(11):2285–2296 10.1007/s10554-011-9889-z PMC3360867 21614485

[5] van Werkhoven JM, Schuijf JD, Gaemperli O (2009). Incremental prognostic value of multi-slice computed tomography coronary angiography over coronary artery calcium scoring in patients with suspected coronary artery disease. Eur Heart J.

[6] Taylor AJ, Cerqueira M, Hodgson JM et al (2010) ACCF/SCCT/ACR/AHA/ASE/ASNC/NASCI/SCAI/SCMR 2010 appropriate use criteria for cardiac computed tomography: a report of the American College of Cardiology Foundation Appropriate Use Criteria Task Force, the Society of Cardiovascular Computed Tomography, the American College of Radiology, the American Heart Association, the American Society of Echocardiography, the American Society of Nuclear Cardiology, the North American Society for Cardiovascular Imaging, the Society for Cardiovascular Angiography and Interventions, and the Society for Cardiovascular Magnetic Resonance. J Am Coll Cardiol 56(22):1864–1894 10.1016/j.jacc.2010.07.005 21087721

[7] de Graaf FR, Schuijf JD, van Velzen JE (2010). Evaluation of contraindications and efficacy of oral Beta blockade before computed tomographic coronary angiography. Am J Cardiol.

[8] de Graaf FR, van Werkhoven JM, van Velzen JE (2010). Incremental prognostic value of left ventricular function analysis over non-invasive coronary angiography with multidetector computed tomography. J Nucl Cardiol.

[9] Schuijf JD, Pundziute G, Jukema JW (2006). Diagnostic accuracy of 64-slice multislice computed tomography in the noninvasive evaluation of significant coronary artery disease. Am J Cardiol.

[10] Schuijf JD, Wijns W, Jukema JW (2006). Relationship between noninvasive coronary angiography with multi-slice computed tomography and myocardial perfusion imaging. J Am Coll Cardiol.

[11] de Graaf FR, Schuijf JD, van Velzen JE (2010). Diagnostic accuracy of 320-row multidetector computed tomography coronary angiography to noninvasively assess in-stent restenosis. Invest Radiol.

[12] Raff GL, Abidov A, Achenbach S (2009). SCCT guidelines for the interpretation and reporting of coronary computed tomographic angiography. J Cardiovasc Comput Tomogr.

[13] de Graaf FR, Schuijf JD, van Velzen JE (2010). Diagnostic accuracy of 320-row multidetector computed tomography coronary angiography in the non-invasive evaluation of significant coronary artery disease. Eur Heart J.

[14] Meijboom WB, Meijs MF, Schuijf JD (2008). Diagnostic accuracy of 64-slice computed tomography coronary angiography: a prospective, multicenter, multivendor study. J Am Coll Cardiol.

[15] Henneman MM, Schuijf JD, van Werkhoven JM (2008). Multi-slice computed tomography coronary angiography for ruling out suspected coronary artery disease: what is the prevalence of a normal study in a general clinical population?. Eur Heart J.

[16] Chow BJ, Abraham A, Wells GA (2009). Diagnostic accuracy and impact of computed tomographic coronary angiography on utilization of invasive coronary angiography. Circ Cardiovasc Imaging.

[17] Hulten EA, Carbonaro S, Petrillo SP (2011). Prognostic value of cardiac computed tomography angiography: a systematic review and meta-analysis. J Am Coll Cardiol.

[18] Bateman TM, O’Keefe JH, Dong VM (1995). Coronary angiographic rates after stress single-photon emission computed tomographic scintigraphy. J Nucl Cardiol.

[19] Shaw LJ, Hachamovitch R, Berman DS (1999). The economic consequences of available diagnostic and prognostic strategies for the evaluation of stable angina patients: an observational assessment of the value of precatheterization ischemia. Economics of noninvasive diagnosis (END) multicenter study group. J Am Coll Cardiol.

[20] Wijns W, De BB, Vanhoenacker PK (2007). What does the clinical cardiologist need from noninvasive cardiac imaging: is it time to adjust practices to meet evolving demands?. J Nucl Cardiol.

[21] Nucifora G, Schuijf JD, van Werkhoven JM et al. (2010) Relationship between obstructive coronary artery disease and abnormal stress testing in patients with paroxysmal or persistent atrial fibrillation. Int J Cardiovasc Imaging 27(6):777–785 10.1007/s10554-010-9725-x PMC3144360 20953841

[22] van Werkhoven JM, Schuijf JD, Gaemperli O (2009). Prognostic value of multislice computed tomography and gated single-photon emission computed tomography in patients with suspected coronary artery disease. J Am Coll Cardiol.

[23] Priest VL, Scuffham PA, Hachamovitch R (2011). Cost-effectiveness of coronary computed tomography and cardiac stress imaging in the emergency department: a decision analytic model comparing diagnostic strategies for chest pain in patients at low risk of acute coronary syndromes. JACC Cardiovasc Imaging.

[24] Hoilund-Carlsen PF, Johansen A, Christensen HW (2006). Potential impact of myocardial perfusion scintigraphy as gatekeeper for invasive examination and treatment in patients with stable angina pectoris: observational study without post-test referral bias. Eur Heart J.

[25] Budoff MJ, Karwasky R, Ahmadi N (2009). Cost-effectiveness of multidetector computed tomography compared with myocardial perfusion imaging as gatekeeper to invasive coronary angiography in asymptomatic firefighters with positive treadmill tests. J Cardiovasc Comput Tomogr.

[26] Achenbach S, Daniel WG (2010). Cardiac imaging in the patient with chest pain: coronary CT angiography. Heart.

[27] Bluemke DA, Achenbach S, Budoff M (2008). Noninvasive coronary artery imaging: magnetic resonance angiography and multidetector computed tomography angiography: a scientific statement from the american heart association committee on cardiovascular imaging and intervention of the council on cardiovascular radiology and intervention, and the councils on clinical cardiology and cardiovascular disease in the young. Circulation.

[28] de Graaf FR, Schuijf JD, Scholte AJ (2010). Usefulness of hypertriglyceridemic waist phenotype in type 2 diabetes mellitus to predict the presence of coronary artery disease as assessed by computed tomographic coronary angiography. Am J Cardiol.

[29] van der Wall EE, Jukema JW, Schuijf JD (2011). 100 kV versus 120 kV: effective reduction in radiation dose?. Int J Cardiovasc Imaging.

[30] Ko BS, Cameron JD, Meredith IT et al. (2011) Computed tomography stress myocardial perfusion imaging in patients considered for revascularization: a comparison with fractional flow reserve. Eur Heart J 33(1):67–77 10.1093/eurheartj/ehr268 21810860

